# Theoretical study of the catalytic performance of Fe and Cu single-atom catalysts supported on Mo_2_C toward the reverse water–gas shift reaction

**DOI:** 10.3389/fchem.2023.1144189

**Published:** 2023-03-20

**Authors:** Wenjuan Zhang, Anna Vidal-López, Aleix Comas-Vives

**Affiliations:** ^1^ Department of Chemistry, Universitat Autònoma de Barcelona, Catalonia, Spain; ^2^ Institute of Materials Chemistry, Technische Universität Wien, Vienna, Austria

**Keywords:** reverse water–gas shift (RWGS) reaction, single-atom catalysis (SAC), Cu/Mo_2_C, Fe/Mo_2_C, DFT calculations

## Abstract

The reverse water–gas shift (RWGS) is an attractive process using CO_2_ as a chemical feedstock. Single-atom catalysts (SACs) exhibit high catalytic activity in several reactions, maximizing the metal use and enabling easier tuning by rational design than heterogeneous catalysts based on metal nanoparticles. In this study, we evaluate, using DFT calculations, the RWGS mechanism catalyzed by SACs based on Cu and Fe supported on Mo_2_C, which is also an active RWGS catalyst on its own. While Cu/Mo_2_C showed more feasible energy barriers toward CO formation, Fe/Mo_2_C presented lower energy barriers for H_2_O formation. Overall, the study showcases the difference in reactivity between both metals, evaluating the impact of oxygen coverage and suggesting Fe/Mo_2_C as a potentially active RWGS catalyst based on theoretical calculations.

## 1 Introduction

As populations and living standards increase, so does our consumption of fossil fuels, coal, oil-derived combustibles, and natural gas. These energy sources eventually transform their carbon content into carbon dioxide (CO_2_), a significant greenhouse gas contributing to global warming and climate change (Karl and Trenberth, 2003; Olah et al., 2011; Lim, 2015; Rodriguez et al., 2015). Consequently, capturing CO_2_ and converting it into fuels and commodity chemicals have attracted considerable attention to mitigate their adverse environmental effects on Earth (Zhang et al., 2019). The reverse water–gas shift (RWGS) reaction (Eq. (1)) is a promising CO_2_ utilization and capture technology because its product can be used directly as feedstock in the Fischer–Tropsch (FT) process, MeOH synthesis processes, and other syngas processes (Guharoy et al., 2019; Jing et al., 2019; Zhang et al., 2019).

$${CO}_{2}+H_{2}↔CO+H_{2}O\;∆H_{298\;K}=41.2\;kJ\;{mol}^{-1}.$$ (1)

Due to the importance of RWGS from both points of view, considerable attention is being paid to improving the reaction kinetic fundamental and practical aspects and designing more efficient RWGS catalysts (Wang et al., 2011; Guharoy et al., 2019). Metal-based catalysts for the RWGS reaction are based on supported particles (Wang et al., 2011). Nevertheless, an emerging class of catalysts enabling optimal metal utilization is single-atom catalysts (SACs) (Qiao et al., 2011; Lin et al., 2013; Wei et al., 2014; Yang et al., 2015; Li et al., 2016; Wang et al., 2016; Lu et al., 2018; Mondelli et al., 2018; Li et al., 2020a; Li et al., 2020b; Kaiser et al., 2020; Gao et al., 2021; Xiong et al., 2021; Zhu et al., 2021). SACs are based on an isolated metal atom anchored on a solid support. Several studies have shown that SACs can exhibit superior catalytic performance in thermocatalytic processes, such as selective hydrogenation (Wei et al., 2014; Wang et al., 2016), CO oxidation (Qiao et al., 2011; Lu et al., 2018), CO_2_ conversion (Li et al., 2020a; Zhu et al., 2021), and water gas-shift (WGS) and RWGS reactions, C–C coupling, and electrocatalytic and photocatalytic processes (Wang et al., 2016; Mondelli et al., 2018; Kaiser et al., 2020) (Lin et al., 2013; Yang et al., 2015; Li et al., 2020b), with high activity, selectivity, metal atom utilization, and stability (Li et al., 2016; Gao et al., 2021; Xiong et al., 2021). For example, Lin et al. (2013) synthesized Ir/FeO_x_ SAC, having exceptionally high activity for WGS, where the Ir center greatly enhanced the reducibility of the FeO_x_ support by generating oxygen vacancies, leading to the excellent catalytic performance. Currently, the development of SACs is a highly active research field (Zhang et al., 2018). Other metal-based catalysts are also promising candidates for the RWGS reaction (Kim et al., 2015; Juneau et al., 2020). Nevertheless, they have drawbacks, that is, their poor natural abundance and high cost. Other alternative materials have also been considered as possible catalysts (Kim et al., 2015). In this context, MXene materials, a family of two-dimensional (2D) carbides, nitrides, and carbonitrides with the general formula of M_n+1_X_n_T_x_ (where M is an early transition metal; n = 1, 2, and 3; X is C; and/or N and T are surface –O–, –OH, and/or –F groups), are currently emerging in thermocatalytic applications as catalysts or supports with reactive metal–support interactions (Li et al., 2018a; Li et al., 2018b; Diao et al., 2018; Zhao et al., 2019; Kurlov et al., 2020). As a member of MXene materials, transition metal carbides (TMCs) have attracted particular attention (Reddy et al., 2019; Lin et al., 2021) as they are cheap, potentially selective, and efficient catalysts.

TMCs have similar properties as precious metals (Zhang et al., 2020; Morales-Salvador et al., 2021), being active in many reactions, such as CO hydrogenation, water–gas shift (WGS), hydrogen evolution reaction (HER), oxygen reduction reaction (ORR), methanol oxidation reaction, and methane reforming (Chen et al., 2016; Wang et al., 2020). As a key member of TMCs, Mo_2_C is particularly interesting for CO_2_ conversion because of its low cost, dual functionality for H_2_ dissociation, and C=O bond scission capability (Porosoff et al., 2014). Many studies have shown that Mo_2_C is highly active in activating CO_2_ in various processes, especially for RWGS reactions. Therefore, combining enriched SACs with Mo_2_C as support is an appealing way to balance catalytic activity, selectivity, and stability effectively (Wang et al., 2022). Theoretical calculations can provide detailed insights into the energetics of the catalytic processes (Geiger and López, 2022). The catalytic cycle, energy barriers catalytic sites, and obtained structure–reactivity relationships of each elementary step can be calculated using DFT-based methods with a good compromise between accuracy and computational cost. Mo_2_C has also been used as a catalyst as the oxygen coverage was a key aspect determining the catalytic activity of the material toward the dry reforming of methane, another CO_2_ conversion process (Kurlov et al., 2020). However, the effect of changing the oxygen coverage on the catalytic activity was not evaluated in depth. In our previous work, we found that Cu SACs on Mo_2_C are highly active catalysts toward the CO_2_ hydrogenation to methanol, showing higher catalytic activity than that of unsupported Cu and Cu/ZnO catalysts. We found that a Cu SAC supported on Mo_2_C and surrounded by O has a high cationic character in agreement with the experiment (Zhou et al., 2021). We proposed feasible reaction mechanisms for the CO_2_ hydrogenation and the RWGS reaction.

In the present article, we address, using theory, that is, DFT calculations, the study of the RWGS reaction catalyzed by SACs supported on TMC (Mo_2_C) with different surface O coverages, particularly Cu- and Fe-based SACs. We focus on the CO_2_ activation and the H_2_O formation, which involve the adsorption of reactants, direct CO_2_ dissociation through the redox mechanism (CO_2_* → CO* + O*), H_2_ dissociation (H_2_* → H* + H*), and water formation (2H* + O* → H_2_O*) (Alonso et al., 2021).

## 2 Computational details

We studied Fe’s and Cu’s catalytic performance supported on Mo_2_CT_x_ with different oxygen coverages, which we denote as Fe/Mo_2_C and Cu/Mo_2_C, respectively. Spin-polarized density functional theory was used for the energetics as implemented in the Vienna Ab initio Simulation Package (VASP) (Kresse and Hafner, 1993; Kresse and Furthmüller, 1996a; Kresse and Furthmüller, 1996b). We used the BEEF-vdW (Wellendorff et al., 2012) as the exchange-correlation functional and projected-augmented wave (PAW)-based pseudopotentials for all calculations. A plane-wave basis set with the kinetic energy cutoff of 500 eV was employed to expand the wave functions. We set the convergence criteria for minima calculations to have a lower force than 0.02 eV/A. A vacuum layer of 10 Å, which is perpendicular to the surface of Fe/Mo_2_C and Cu/Mo_2_C, was added to avoid spurious interactions between periodic images. For gas-phase calculations of molecules, we employed a cubic supercell of 15 Å × 15 Å × 15 Å. We included dipole corrections along the *z*-direction due to the asymmetry of the M/Mo_2_C surface with co-adsorbed oxygen atoms. We used nudged-elastic band (NEB) methods to locate the transition states until the atomic forces were less than 0.05 eV/Å. Finally, we constructed the energy profile for the RWGS for all evaluated systems referencing all minima and transition states against the sum of energies of the given evaluated catalyst and initial reactants (CO_2_ and H_2_) as the origin of energies.

## 3 Results and discussion

**FIGURE 1:**
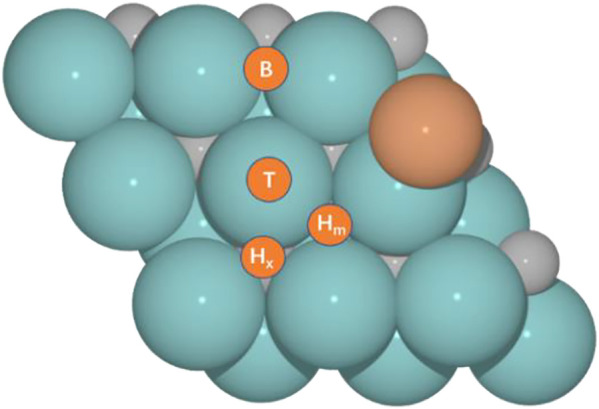
Optimized structures for M (Cu and Fe)/Mo_2_C catalytic systems and four adsorption sites, namely, top (T), bridge (B), and hollow sites at X atom (H_x_) or metal (H_m_).

We selected our former model for the Cu/Mo_2_CT_x_ system (Zhou et al., 2021), hereafter Cu/Mo_2_C, to evaluate their activity toward the RWGS reaction for different oxygen coverages (O ML); 0, 0.33, 0.67, and 0.78, and performed an analogous study for the hypothetical Fe/Mo_2_C one. Figure 1 shows the structures of Fe/Mo_2_C and Cu/Mo_2_C. CO_2_ and H_2_ adsorption minima close in energy were considered as initial structures. From their most stable configurations, other minimums and transition states were localized. For each adsorbate (CO_2_, CO, O, and H), four high-symmetry sites were explored (Figure 1), namely, top (T), bridge (B), and two types of threefold hollow sites, either with an X atom (H_x_) or a metal (H_m_) atom beneath. The adsorption energy for each adsorbate (CO_2_, CO, O, and H) on each site for both systems is provided in Supplementary Table S1 of the Supporting Information.

### 3.1 Description of the reverse water–gas shift reaction mechanism

**FIGURE 2:**
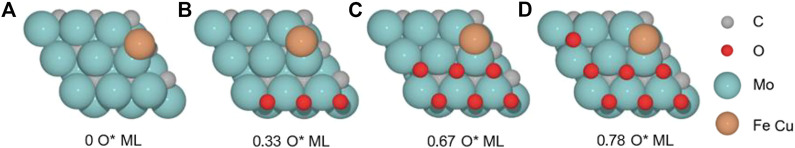
Top and side views of the structure of M/Mo_2_C catalyst (M = Cu and Fe) with **(A)** 0 ML, **(B)** 0.33 ML, **(C)** 0.67 ML, and **(D)** 0.78 ML surface oxygen coverages. Gray, cyan, red, and bronze spheres indicate carbon (C), molybdenum (Mo), oxygen (O), and metal (Cu and Fe) atoms, respectively.

We studied the RWGS reaction catalyzed by the M(Cu and Fe)/Mo_2_C system, which we previously evaluated for a 0.67 O ML coverage as a side reaction of the CO_2_ hydrogenation to methanol reaction for the Cu/Mo_2_C system (Zhou et al., 2021; Geiger and López, 2022). In the present work, we systematically assess the oxygen coverage’s effect on the energetics of the RWGS reaction for Cu and Fe SACs supported on Mo_2_C. Thus, we first evaluated the clean M/Mo_2_C system, that is, without oxygen being adsorbed, and 0.33, 0.67, and 0.78 O ML systems. We optimized the system’s minima and transition states to evaluate the RWGS mechanism for both catalysts at several oxygen coverages to assess the latter’s effect and compare the intrinsic activity of Fe and Cu on the RWGS activity (Figure 2 **)**.

**FIGURE 3:**
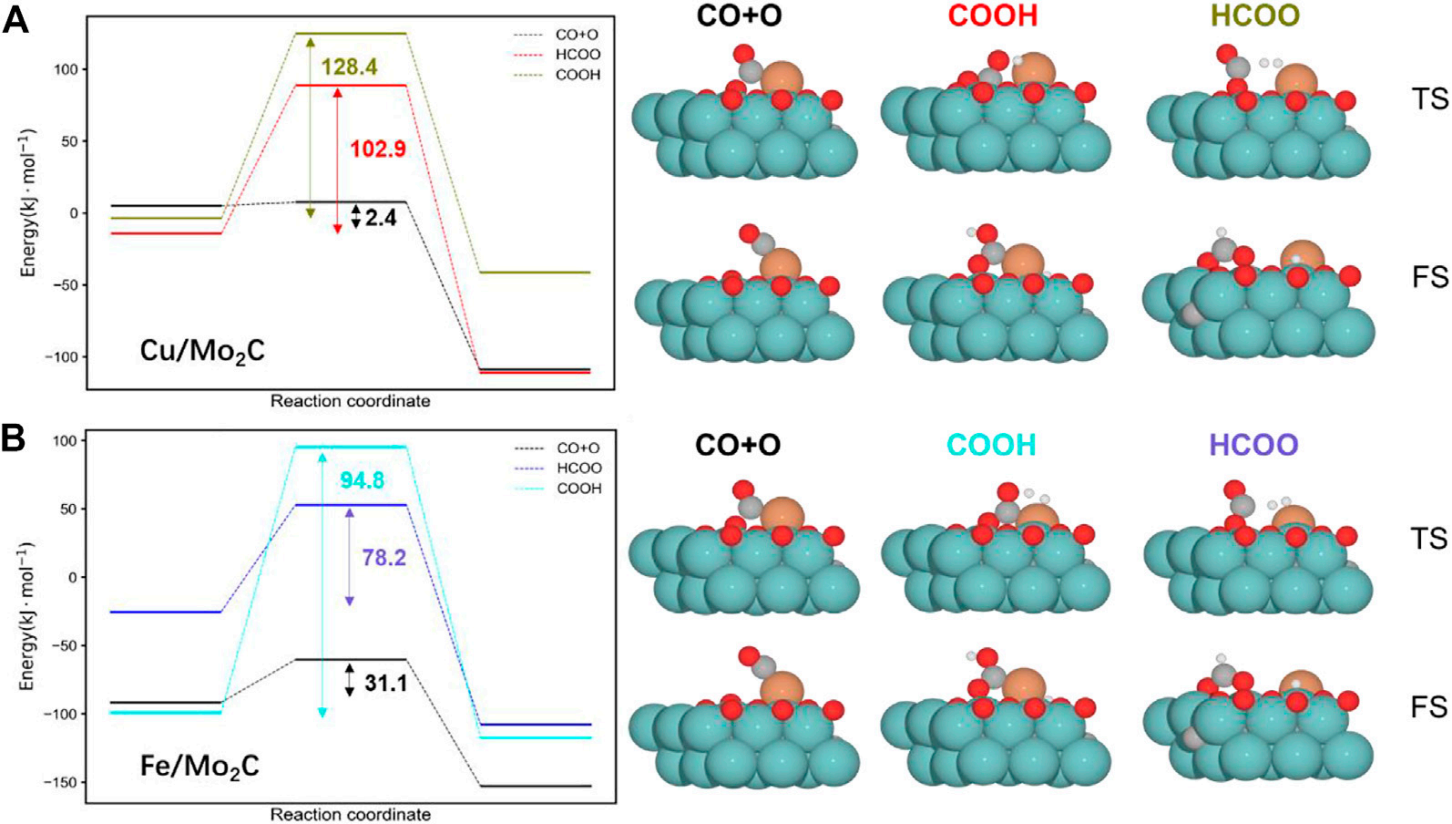
Energy profiles for the Cu/Mo_2_C **(A)** and Fe/Mo_2_C **(B)** systems comparing the direct CO_2_ cleavage (CO + O), that is, the redox pathway and the formation of formate (HCOO) and carbonyl (COOH) intermediates. The energy barriers are reported within each profile (black indicates the lowest pathway for each system). The optimized structures for the transition state (TS) and final state (FS) structures are shown next to the corresponding energy profiles. Energies are referenced against the sum of the initial reactants’ energy in kJ mol^–1^ (E_rel_).

We split the RWGS reaction into two key steps, namely, CO_2_ activation (CO*+O*) and water formation (H_2_O*). Concerning CO_2_ activation, hydrogen-assisted routes *via* formate (HCOO*) and carbonyl (COOH*) are an alternative to direct CO_2_ activation. Nevertheless, forming HCOO* and COOH* species for both evaluated catalysts is more demanding than just directly splitting CO_2_ (see Figure 3). Thus, assessing the subsequent C–O bond cleavage of HCOO* and COOH* is not needed to conclude that the redox pathway by direct activation of CO_2_ is preferred over the hydrogen-assisted routes.

We will now describe in detail the CO_2_ activation step. First, the CO_2_ molecule adsorbs on the metal atom (M = Cu, Fe)/Mo_2_C interface, forming a *δ*–CO_2_* intermediate. Subsequent CO_2_ pre-activation is exoenergetic or slightly endoenergetic, depending on the oxygen coverage. In the resulting structure, the carbon atom and one oxygen atom from CO_2_ carbon bind directly to the metal center, while the second oxygen of CO_2_ coordinates to a Mo atom. From the *δ*–CO_2_* structure, CO_2_ can split *via* TS1 into CO* and O* in an endoenergetic step for all the cases. This transition state allows activating CO_2_ and cleaving one of the C–O bonds. Oxygen bonds on the highly oxophilic Mo-hollow sites, while CO* remains coordinated to the metal center (M = Cu/Fe). The next step we evaluated is the desorption of the CO molecule to the gas phase. This step is endoenergetic for all cases. Given the high temperature of the RWGS reaction (200°C–500 °C for maximum conversion of CO_2_ ranging from 10% to 50%) (Porosoff et al., 2016), both the CO_2_ cleavage and the CO* desorption seem feasible at both the kinetic and thermodynamic levels.

The second part of the mechanism corresponds to the H_2_O molecule formation. This process is endoenergetic in all cases. This pathway starts with the adsorption of the H_2_ molecule on the surface, which is exoenergetic in all cases. Next, the H–H bond cleaves (TS2), giving rise to a proton (H^+^) and a hydride ion (H^−^). The latter transition state can be understood as a heterolytic TS, producing formally a metal hydride (M–H) and the proton bonded to the cleaved O*. The resulting formal metal hydride remains at the interface (H*–M/Mo_2_C) and the hydroxy group on a Mo-hollow site (HO*–Mo). The subsequent migration of the H* to the OH* group and the O–H bond formation to produce the H_2_O* molecule has a high energy barrier (TS3). This transition state is the most energy demanding along the energy profile for all the evaluated oxygen coverages. After forming H_2_O*, its desorption is endoenergetic for all systems.

### 3.2 Fe/Mo_2_C system

**FIGURE 4:**
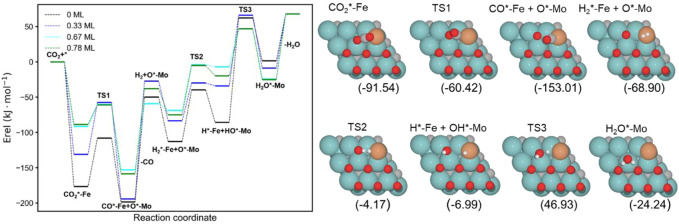
Energy profile of the RWGS reaction on Fe/Mo_2_C with different O ML coverages; black, blue, cyan, and green lines indicate surface oxygen coverages of 0, 0.33, 0.67, and 0.78, respectively. The intermediate and transition state structures and their energies are shown on the right for the 0.67 O ML case. Energies are referenced against the sum of the initial reactants’ energy in kJ mol^–1^ (E_rel_).

The energy profiles of the RWGS catalyzed by Fe/Mo_2_C with 0 ML, 0.33 ML, 0.67 ML, and 0.78 ML surface oxygen coverages are shown in Figure 4. Table 1 summarizes the energy barriers for all evaluated steps.

**TABLE 1:** Summary of energy values (adsorption, release, and overall reaction) and energy barriers (transition states) for each step on RWGS with the Fe/Mo_2_C system in kJ mol^−1^.

Step	Energy values (kJ mol^−1^)	Oxygen coverage (O ML)			
		0	0.33	0.67	0.78
CO	δ–CO_2_ adsorption	−176.6	−131.2	−91.5	−88.5
	∠O–C–O (°)	122.0	132.3	136.1	136.4
	Reaction energy (CO)	−21.9	−63.1	−61.5	−70.1
	CO desorption	148.7	166.9	93.8	120.7
H_2_O	H_2_ adsorption	−62.9	−56.1	−9.8	−37.1
	Reaction energy (OH*+H*-Fe)	27.2	49.8	7.3	17.7
	Reaction energy (H_2_O)	87.2	24.9	37.3	32.2
	H_2_O desorption	66.5	76.7	92.3	93.1
Step	Energy barriers (kJ mol^−1^)	Oxygen coverage (O ML)			
		0	0.33	0.67	0.78
CO	CO_2_ splitting (TS1)	68.7	73.6	31.1	27.4
H_2_O	H_2_ splitting (TS2)	73.2	53.6	64.7	69.8
	H_2_O formation (TS3)	147.7	100.4	53.9	66.6

**FIGURE 5:**
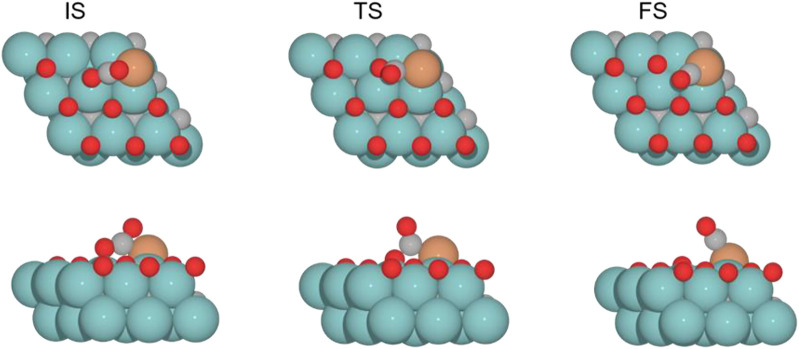
Top and side views of the initial state (IS), transition state (TS), and final state (FS) for the CO_2_ cleavage catalyzed by the Fe/Mo_2_C of 0.78 ML system (see Supplementary Figures S1–S3 for the structures of other intermediates and TS on 0, 0.33, and 0.67 O* ML coverages).

The adsorption of CO_2_ is exoenergetic for all coverages, by 176.6, 131.2, 91.5, and 88.5 kJ mol^–1^ going from lower to higher oxygen coverages. All adsorbed CO_2_ molecules have a bent structure, in which one oxygen is bound to the Fe center, while the other one is bound to a Mo-top site, and the carbon atom is bonded to the Fe and the two Mo-top sites, as shown in the initial state (IS) on Figure 5. The oxygen coverage effect can explain the energy differences in CO_2_ pre-activation. On the one hand, lower oxygen coverage means less repulsion between the adsorbed surface species and the catalytic system. Thus, higher CO_2_ adsorption energies are obtained for the 0 and 0.33 O ML systems shown in Table 1. Overall, the *δ*–CO_2_* intermediate is more stable when decreasing the oxygen coverage. On the other hand, the resulting bent CO_2_ angles of the resulting intermediate (∠O–C–O) are 122° (0 ML), 132.3° (0.33 ML), 136.1° (0.67 ML), and 136.4° (0.78 ML), confirming the relationship between the bending angle and the energy gain upon adsorbing CO_2_ on the catalytic surface.

The subsequent CO_2_ cleavage step (TS1) has energy barriers equal to 68.7, 73.6, 31.5, and 27.4 kJ mol^–1^, from lower to higher oxygen coverages. These energy barriers are related to the *δ*–CO_2_ pre-activation and stability. The energy barriers for CO_2_ cleavage slightly decrease when increasing the ∠O–C–O angle and decreasing the energy stability of the *δ*–CO_2_* intermediate. Overall, reaction energy differences are exoenergetic, so all catalysts are favorable for the formation of CO, as shown in Table 1. The desorption of the resulting CO* species on Fe (FS; Figure 5) is endoenergetic in all cases, that is, by 148.7, 166.9, 93.8, and 120.7 kJ mol^–1^ (see Figure 4). For the first two coverages (0 and 0.33 ML), the energy for the CO* desorption includes slight Fe movement (Supporting Information Supplementary Table S2 reports the energy difference involved in both Fe displacements). The 0 ML coverage has the most favorable adsorption energy, confirming a more significant interaction between CO_2_ with Mo sites and the iron center. Among all the coverages evaluated, 0.67 ML has a lower CO release energy, but 0.78 ML allows a better rate of CO formation due to its affordable reaction barrier and moderate releasing energy.

**FIGURE 6:**
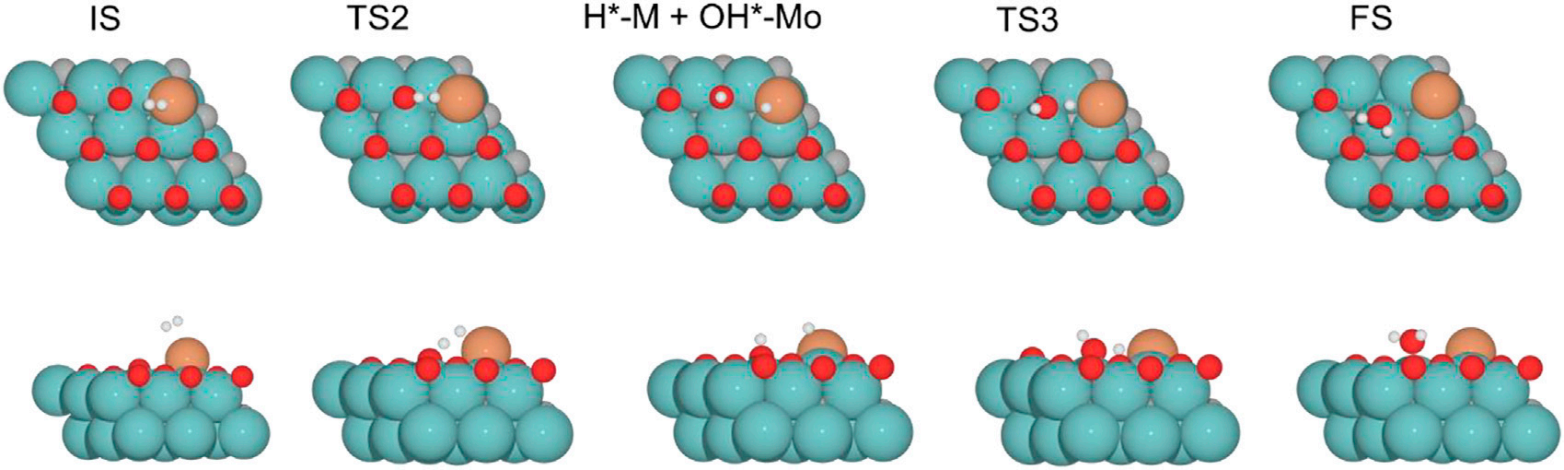
Top and side views of structures of initial state (IS), intermediate, transition state (TS), and final state (FS) on Fe/Mo_2_C of 0.78 O ML coverage for H_2_ splitting and the formation of H_2_O (see Supplementary Figures S4-6 for the structures of other intermediates and TS on 0, 0.33, and 0.67 O* ML coverages).

The H_2_O formation starts *via* H_2_ adsorption, which is exoenergetic in all cases. The adsorption energies are 63.2, 56.4, 9.8, and 37.3 kJ mol^–1^ for oxygen coverages equal to 0, 0.33, 0.67, and 0.78 ML, respectively. Again, the 0 O ML system has the most favorable adsorption energy, in which the location of the H_2_ molecule coordinates to Fe but is closer to the co-adsorbed oxygen coming from the CO_2_ activation than the other oxygen coverages just above the Fe, and the co-adsorbed oxygen favors the adsorption. It means that the more in the middle it is, the better the energy absorption. The energy barriers for the subsequent heterolytic H_2_ cleavage (TS2, Figure 6) are equal to 73.2, 53.4, 64.7, and 69.8 kJ mol^–1^ for 0, 0.33, 0.67, and 0.78 ML coverages, respectively. The H_2_ cleavage forms an OH* species adsorbed on a Mo-hollow site and a metal hydride intermediate (H*–M + OH*–Mo, Figure 6).

Finally, H_2_O forms by reaction of OH* and H* with energy barriers equal to 147.7, 100.4, 53.9, and 66.6 kJ mol^−1^ for oxygen coverages of 0, 0.33, 0.67, and 0.78 ML, respectively. TS3 geometries differ only in the proximity of the H_2_O* formed to the Mo-hollow and in the migration step of the H* atom from the interface to the OH* group (TS3, Figure 6). The position of the OH* group, the migration site of the H* atom, and the bond lengths Fe–H and H–OH on the TS3 differ depending on the oxygen coverage, as is summarized in Table 2.

**TABLE 2:** Summary of OH* position, H* migration’s site, and H–Fe and H–OH bond lengths for the H* atom migration step from the Fe/Mo_2_C interface to OH* involved in the H_2_O* formation step (TS3) of the RWGS catalyzed by the Fe/Mo_2_C system in Å.

Surf.—Cover. (ML)	OH* position	H* migration site	H–Fe (Å)	H–OH (Å)
0	Mo-top	Interface	1.85	1.37
0.33	Mo-top	Mo-hollow	3.08	1.38
0.67	Mo-hollow	Fe-top	1.95	1.29
0.78	Mo-hollow	Fe-top	1.75	1.40

**FIGURE 7:**
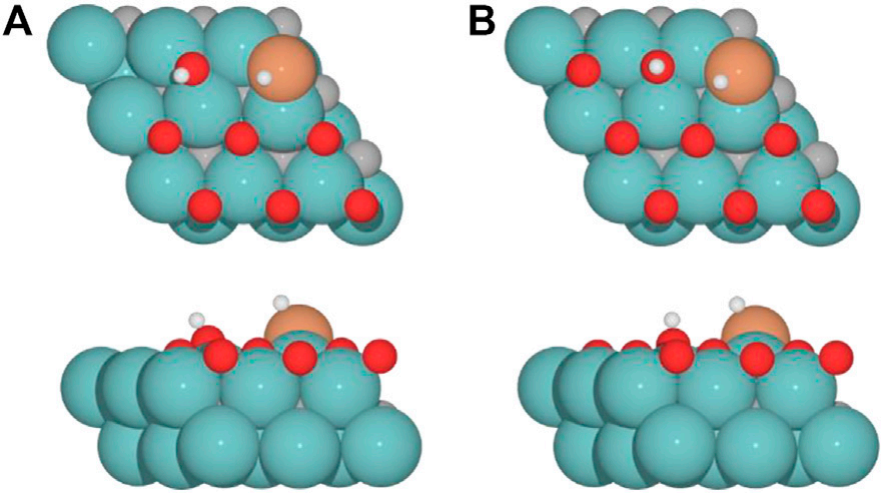
Top and side views of new minima were obtained where the H* atom remains at the top of the metal center involved in Fe/Mo_2_C coverage of **(A)** 0.67 and **(B)** 0.78 ML for H_2_O* formation.

For the clean Fe/Mo_2_C system, the H* atom comes from the interface, while the OH* group has more available adsorption sites as there is no oxygen around. At 0.33 O ML, the H* atom goes to Mo-hollow because it is available in the absence of further oxygen coverage. Finally, for 0.67 and 0.78 ML, there are one or no longer any adsorption sites available, and consequently, the H* atom moves to the metal atom instead of the Fe/Mo_2_C interface before forming H_2_O*. These newly obtained minima, in which H* is solely bonded to the metal center, are shown in Figure 7. In these final cases, the OH* group remains bonded to the Mo-hollow adsorption site. Structures of initial state (IS), transition state (TS), intermediate, and final state (FS) are shown in Figure 6.

The H_2_O* structure at 0.67 and 0.78 O ML, that is, with the high oxygen coverage, is more stable due to the formation of two hydrogen bonds between H_2_O* and a co-adsorbed oxygen atom (FS, Figure 6). The desorption of H_2_O is endoenergetic for all systems by 66.5, 76.7, 92.3, and 93.1 kJ mol^–1^, from lower to higher oxygen coverages. High oxygen coverages, likely present under reaction conditions, provide the most feasible energy barriers, and therefore, high catalytic activity is expected. In contrast, for low oxygen coverages, the strong adsorption of the intermediates increases the key energy barriers for the RWGS reaction, suggesting a lower catalytic activity. The most active system along the evaluated series is the Fe/Mo_2_C surface with a 0.67 O ML coverage as it presents the lowest energy barrier, with the highest energy barrier being the H_2_O formation step, amounting to 53.9 kJ mol^-1^.

### 3.3 Cu/Mo_2_C system

**FIGURE 8:**
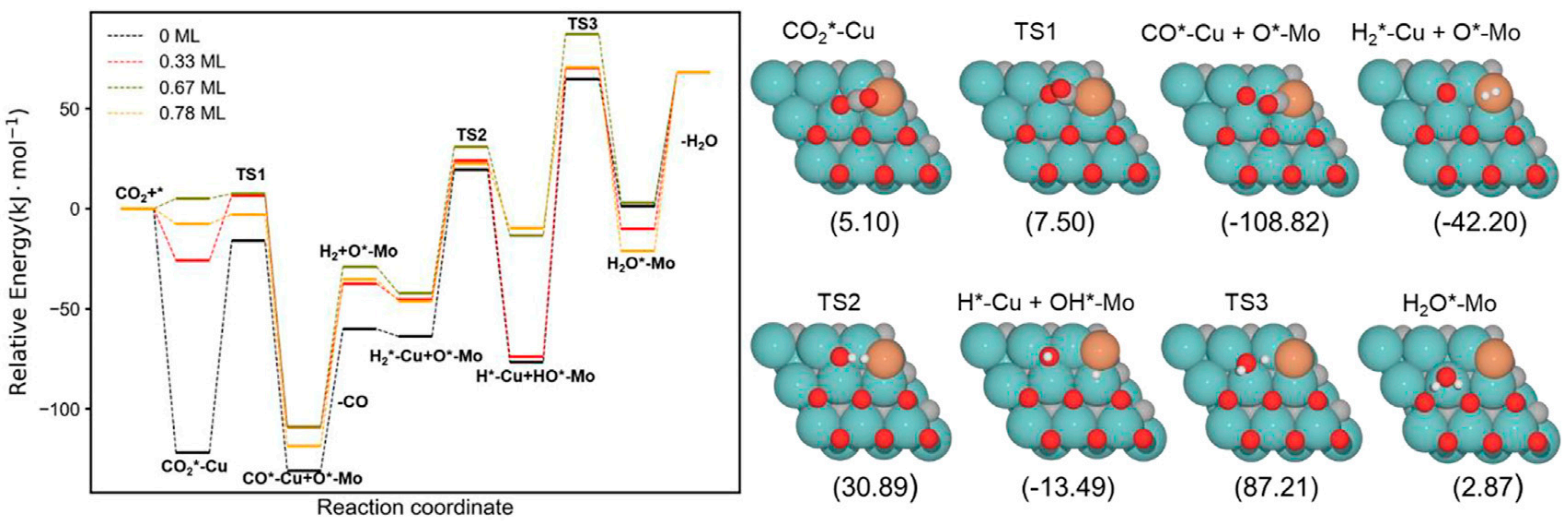
Energy profile of RWGS reaction on Cu/Mo_2_C with different O ML coverage; black, red, green, and orange lines indicate surface oxygen coverages of 0, 0.33, 0.67, and 0.78, respectively. The intermediate and transition state structures and their energies are shown on the right for the 0.67 O ML case. Energies are referenced against the sum of the initial reactants’ energy in kJ mol^–1^ (E_rel_).

Figure 8 shows the complete energy profile of the RWGS catalyzed by the Cu/Mo_2_C with 0 ML, 0.33 ML, 0.67 ML, and 0.78 ML oxygen coverages**.** The energy barriers of each step are summarized in Table 3 **.**

**TABLE 3:** Summary of energy values (adsorption, release, and overall reaction) and energy barriers (transition states) for each step on RWGS with Cu/Mo_2_C system in kJ mol^−1^.

Step	Energy values (kJ mol^−1^)	Oxygen coverage (O ML)			
		0	0.33	0.67	0.78
CO	δ–CO_2_ adsorption	−121.9	−25.9	5.1	−7.6
	∠O–C–O (°)	121.4	133.8	136	139
	Reaction energy (CO)	−9.1	−83.2	−103.4	−110.9
	CO desorption	70.9	71.6	79.9	83.2
H_2_O	H_2_ adsorption	−3.7	−7.9	−13.9	−10.8
	Reaction energy (OH*+H*–Cu)	−12.8	−28.6	28.7	36.6
	Reaction energy (H_2_O)	77.8	63.8	16.4	−11.4
	H_2_O desorption	66.8	78.1	65.1	89.1
Step	Energy barriers (kJ mol^−1^)	Oxygen coverage (O ML)			
		0	0.33	0.67	0.78
CO	CO_2_ splitting (TS1)	105.8	32.4	2.4	4.7
H_2_O	H_2_ splitting (TS2)	83.2	69.3	73.1	68.8
	H_2_O formation (TS3)	141.4	144.1	100.7	80.2

**FIGURE 9:**
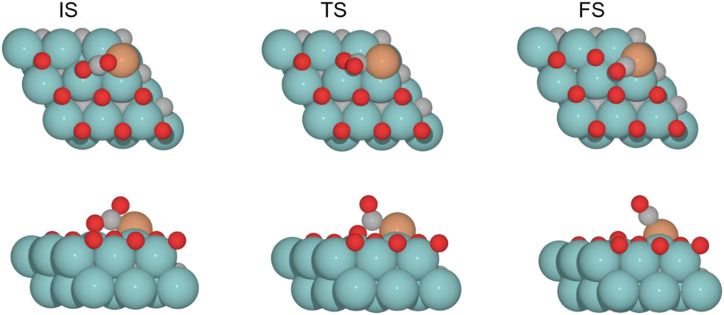
Top and side views of the initial state (IS), transition state (TS), and final state (FS) catalyzed by the Cu/Mo_2_C of 0.78 ML system for the CO_2_ cleavage (see Supplementary Figures S7-9 for the structures of other intermediates and TS on 0, 0.33, and 0.67 O* ML coverages).

The CO_2_ adsorption is exoenergetic by 121.9, 25.9, and 7.6 kJ mol^–1^ for 0 , 0.33 , and 0.78 ML coverages, respectively, while for 0.67 ML, it is slightly endothermic, which is about 5.1 kJ mol^–1^. On the Cu/Mo_2_C catalyst, a low oxygen coverage decreases the repulsion of the adsorbed CO_2_ and therefore results in a more favorable CO_2_ adsorption energy. For Cu/Mo_2_C 0 ML, CO_2_ binds mainly on Mo rather than on Cu in comparison to the other coverages, in a very exothermic adsorption step of 121.9 kJ mol^–1^, as mentioned earlier (CO_2_*–Cu, Figure 8). In this structure, CO_2_ bends the most, with an ∠O–C–O angle equal to 121.4°. For the rest coverages (0.33, 0.67, and 0.78 ML), the carbon and one oxygen atom of CO_2_ are bonded to the Cu center, and the oxygen of CO_2_ is connected to the top Mo site (shown as IS in Figure 9).

**FIGURE 10:**
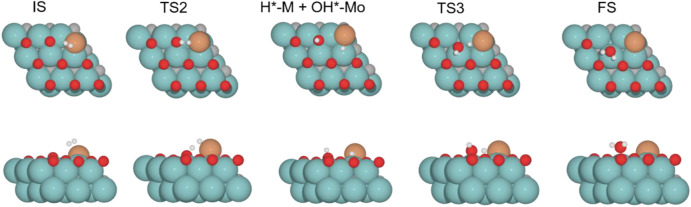
Top and side views of structures of the initial state (IS), intermediate, transition state (TS), and final state (FS) on Cu/Mo_2_C of 0.78 O ML coverage for H_2_ splitting and the formation of H_2_O (see Supplementary Figures S10-12 for the structures of other intermediates and TS on 0, 0.33, and 0.67 O* ML coverages).

The energy barriers for the subsequent CO_2_ splitting (TS1) are 105.8, 32.4, 2.4, and 4.7 kJ mol^–1^. As found for the Fe/Mo_2_C system (*vide supra*), a more stable *δ*–CO_2_ intermediate implies a high energy barrier for CO_2_ cleavage; that is, a high oxygen coverage favors CO_2_ activation. Overall, the reaction energies are exoenergetic, so all catalysts favorably form CO, as shown in Table 3. Once CO* is obtained, the CO* desorption is endoenergetic in all the cases, arising from the strong bond between CO* and the Cu atom, as we can see in the optimized minimum CO*–Cu + O*–Mo shown in Figure 8 and as FS in Figure 9. The 0 ML coverage has the most significant adsorption energy and the highest reaction barrier for CO_2_ activation, suggesting a more substantial interaction between the CO_2_ and the catalyst increases the energy barrier. In contrast, 0.78 ML has the highest CO desorption energy (83.2 kJ mol^-1^), but all oxygen coverages present CO desorption values within 70.9–83.2 kJ mol^–1^. When the oxygen coverage is equal to 0.67 O ML, the lowest energy barrier toward CO* + O* is obtained: 2.4 mol^–1^. After CO desorbs, the adsorption of H_2_ is exoenergetic by 3.7, 7.9, 13.9, and 10.8 kJ mol^–1^, from lower to higher oxygen coverages. Next, H_2_ splits in a heterolytic way. The hydride ion (H^−^) remains on the Cu/Mo_2_C interface, while the proton (H^+^) bonds to the O* atom arising from the CO_2_ cleavage, forming an OH* group bonded to Mo, as shown in Figure 10. The reaction barriers for H_2_ splitting (heterolytic TS2, Figure 10) are 83.2, 69.3, 73.1, and 68.8 kJ mol^–1^ for 0 ML, 0.33 ML, 0.67 ML, and 0.78 ML, respectively. We can observe that when the H_2_ adsorption is higher, the energy barrier for the H–H bond cleavage decreases (Table 3).

Finally, the energy barriers to forming H_2_O (TS3, Figure 10) are 141.4, 144.1, 100.7, and 80.2 kJ mol^–1^ from lower to higher oxygen coverages. This step has the highest energy barriers for all evaluated oxygen coverages. The related transition states correspond to the formation of the second O–H bond of water by the hydrogen transfer of the H* atom at the Cu/Mo_2_C interface to the OH* group adsorbed to the Mo-hollow site. The geometries of TS3 are similar for all coverages. The main differences observed are the position of the OH* group, the migration site of the H* atom, and the distance length when migrating from the Cu/Mo_2_C interface to OH*. Table 4 summarizes the related position/migration and the Cu–H and O–H bond lengths for TS3. In this case, the bond length of the H* atom to Cu of the interface allows a higher reactivity of this hydrogen atom, resulting in a lower energy barrier. Structures of initial state (IS), transition state (TS), intermediate, and final state (FS) are shown in Figure 10.

**TABLE 4:** Summary of OH* position, H* migration’s site, and H–Cu and H–OH bond lengths for the H* atom migration step from the Cu/Mo_2_C interface to OH* involved in the H_2_O* formation step (TS3) of the RWGS catalyzed by the Cu/Mo_2_C system in Å.

Surf.—Cover. (ML)	OH* position	H* migration site	H–Cu (Å)	H–OH (Å)
0	Mo-bridge	Interface	2.67	1.26
0.33	Mo-bridge	Interface	1.99	1.30
0.67	Mo-hollow	Interface	1.76	1.38
0.78	Mo-hollow	Interface	1.75	1.36

H_2_O desorption steps are all endoenergetic by 66.8, 78.1, 65.2, and 89.1 kJ mol^–1^ from lower to higher oxygen coverages. These resulting products with the water molecule adsorbed are more stable for high oxygen coverages (0.67 and 0.78 O ML) than for the lower ones (0 and 0.33 O ML) due to the formation of hydrogen bonds between water and the co-adsorbed oxygen atom (FS, Figure 10). The Cu/Mo_2_C system shows that the more favorable the reaction energy, the lower the energy barrier for forming H_2_O.

Overall, among all the RWGS catalyzed by Cu/Mo_2_C, the systems with high oxygen coverages have the lowest energy barriers for CO_2_ activation and H_2_O formation (0.67 and 0.78 O ML) compared to the systems with low oxygen coverages (0 and 0.33 O ML). Overall, the system with the lowest energy barriers is the Cu/Mo_2_C 0.78 O ML one, in which the highest energy barrier is 80.2 kJ mol^-1^, corresponding to the water formation step.

### 3.4 Comparison of the RWGS catalytic activity of Cu/Mo_2_C vs Fe/Mo_2_C

The discussion will be divided into two parts: one for 0 and 0.33 O ML coverages and the other for 0.67 and 0.78 O ML coverages, respectively. We first describe the results for the CO formation with the 0/0.33 O ML systems. The adsorption of CO_2_ releases energy in all cases. When increasing the amount of co-adsorbed oxygen, the (∠O–C–O) angle and the adsorption energy decrease; that is, it is less negative—from −121.9 to −25.9 kJ mol^–1^ for Cu/Mo_2_C and −176.6 to −131.2 kJ mol^–1^ for Fe/Mo_2_C. These values indicate that CO_2_ interaction is significantly stronger on Fe than on Cu on clean surfaces. All reaction energies are exoenergetic for cleaving CO_2_ to CO* and O*. The energy barriers of CO_2_ splitting for Cu are 105.8 and 32.4 kJ mol^–1^ for 0 and 0.33 O ML, respectively, whereas, for Fe, they are equal to 68.7 and 73.6 kJ mol^–1^ for 0 and 0.33 O ML, respectively. The Cu system presents an essential difference between both coverages since the 0.33 O ML coverage has a much lower energy barrier than the 0 ML one: 105.8 vs 32 kJ mol^-1^. In contrast, the Fe system only shows a difference of 5 kJ mol^-1^ between 0 and 0.33 O ML. The Cu/Mo_2_C at 0.33 O ML is the most active system toward cleaving CO_2_. The reaction energy of this step becomes more negative and, therefore, more favorable upon increasing the surface oxygen coverage for both Fe/Mo_2_C and Cu/Mo_2_C. Nevertheless, the variation is more significant for Cu/Mo_2_C, indicating that the presence of surface oxygen atoms substantially affects the Cu/Mo_2_C system more than the Fe/Mo_2_C one. Finally, CO desorption is endothermic for both Fe/Mo_2_C (148.6 and 167 kJ mol^-1^) and Cu/Mo_2_C (70.9 and 71.6 kJ mol^-1^). Here, a remarkable difference between both systems is that the energy required to desorb CO is much higher for Fe/Mo_2_C than for Cu/Mo_2_C, regardless of oxygen coverage. This difference means the CO binding energy is much stronger on Fe/Mo_2_C than on Cu/Mo_2_C. However, at the high temperature of RWGS, desorption is favored entropically, so it should be feasible for both catalysts. Concerning H_2_ adsorption, it is more favorable on the Fe/Mo_2_C catalyst than on the Cu/Mo_2_C one. The Fe system has a maximum energy release of 62.9 kJ mol^–1^ per 0 ML. The splitting of H_2_ to OH*+H*–M is endothermic in Fe, while for Cu, it is exothermic. The reaction energy absorbed for Fe or released for Cu energy increases with coverage; 0.33 ML exhibits better OH* formation for both metals due to the lower energy barrier. The second energy barrier for obtaining H_2_O, both Fe/Mo_2_C and Cu/Mo_2_C, shows the same trend, with the highest energy barriers and endothermic processes. Finally, H_2_O desorption requires similar adsorption energy values on both metal systems and coverages.

Concerning the catalytic performance of Fe/Mo_2_C and Cu/Mo_2_C with surface oxygen coverages of 0.67 ML and 0.78 M O ML, the adsorption of CO_2_ is more favored for Fe/Mo_2_C than for Cu/Mo_2_C. The energy released is less negative upon increasing oxygen content and the ∠O–C–O angle, which is consistent with the behavior from 0ML to 0.3 ML oxygen coverages. However, Cu/Mo_2_C has a slight endothermic reaction at 0.67 ML instead of 0.78 ML, which is exothermic, and the energy released is less than that of Fe/Mo_2_C with the same oxygen coverage. Again, the overall reaction energy differences are exothermic, so the CO formation is thermodynamically favorable for all catalysts. The energy barriers for the CO_2_ splitting at 0.67 and 0.78 O ML are the lowest in both systems and are like each other, although they are lower for the Cu/Mo_2_C systems (2.4–4.7 kJ mol^-1^) than for the Fe/Mo_2_C ones (27.4–31.1 kJ mol^-1^). For CO formation, Fe/Mo_2_C and Cu/Mo_2_C show the same energy trend as oxygen increases, with the highest reaction energy released for the oxygen coverage equal to 0.78 ML. Finally, higher energy was required in the desorption of CO 0.78 ML, with both metals indicating better interaction with CO*. Concerning the subsequent H_2_ adsorption, the Fe/Mo_2_C 0.78 O ML system releases more energy than the Cu one. Fe and Cu systems have negligible energy differences in the energy barriers for H_2_ splitting. The reaction energies to form OH become more endoenergetic as oxygen coverage increases. The energy barriers for H_2_O formation for the oxygen coverages equal to 0.67 and 0.78 ML are lower for the Fe/Mo_2_C than for the Cu/Mo_2_C ones, ranging within 53.9–66.6 kJ mol^-1^ and 80.2–100.7 kJ mol^-1^, respectively. Overall, Fe/Mo_2_C, with 0.67 O ML, has the lowest energy barrier for forming water: 53.9 kJ mol^-1^. Finally, H_2_O desorption requires higher adsorption energy values on Fe/Mo_2_C, indicating a strong interaction between H_2_O and iron instead of copper.

In summary, for CO_2_ and H_2_ adsorption, Fe/Mo_2_C with 0 ML coverage is the most energy-releasing system upon the adsorption of the reactants. For CO formation, Cu/Mo_2_C 0.67 ML has the lowest energy barrier (TS1, 2.4 kJ mol^–1^), and it is among those with the lowest reaction energies (−103.4 kJ mol^–1^). Therefore, it is the system leading most easily to CO. The CO desorption values are higher for Fe catalysts than for Cu ones, indicating a higher interaction of CO on Fe than on Cu/Mo_2_C. Concerning H_2_ splitting, Fe/Mo_2_C 0.33 ML presents the lowest energy barrier (TS2, 53.6 kJ mol^–1^) among all systems. However, the energy barriers for both systems and coverages do not differ much, being all within 53.6–83.2 kJ mol^-1^. Fe/Mo_2_C 0.67 O ML is the system presenting the lowest energy barrier for forming H_2_O (TS3: 53.9 kJ mol^–1^), followed by the Fe/Mo_2_C 0.78 O ML system (66.6 kJ mol^-1^). Conversely, the energy barriers for this transition state (TS3) for 0 and 0.33 O ML for Fe (147.7 and 100.4 kJ mol^-1^, respectively) and Cu (141.1 and 144.1 kJ mol^–1^, respectively) present high energy barriers. According to the overall analysis, the coverage of 0.67 O ML is the most effective one in catalyzing the formation of CO and the formation of H_2_O. While Cu more favorably forms CO and Fe H_2_O, the best Fe/Mo2C system (0.67 O ML) presents the lowest energy barriers.

## 4 Conclusion

Systematic DFT calculations were performed on Cu/Mo_2_C and Fe/Mo_2_C catalysts to explore the effect of metal and different oxygen coverages through the RWGS reaction. The study indicates that both catalysts can pre-activate and cleave CO_2_, heterolytically split H_2_, and form water by reacting with two adsorbed hydrogen atoms, formally as a proton (H^+^) and a hydride ion (H^−^). On the one hand, Cu/Mo_2_C showed more feasible energy barriers for CO formation, while Fe/Mo_2_C presented more feasible energy barriers for H_2_O formation. Overall, the most active Fe/Mo2C system, having oxygen coverage equal to 0.67 O ML, presents lower energy barriers than the Cu/Mo_2_C system, suggesting that it is more active than the latter. The presence of O in the catalysts may explain the previously mentioned favorable trends for high coverages for RWGS reactivity. On the other hand, H_2_ activation has similar trends for both metals and all oxygen coverages. In conclusion, the calculated energy barriers and reaction energies suggest that the Fe/Mo_2_C 0.67 O ML catalyst has the potential for being a highly active RWGS catalyst, likely arising from the highly oxophilic and positive character of Fe, in which the high oxygen coverage balances the catalytic activity in agreement with the Sabatier principle.

## Data Availability

The raw data supporting the conclusions of this article will be made available by the authors, without undue reservation.
