# Supplementary material for: Theoretical study of the catalytic performance of Fe and Cu single-atom catalysts supported on Mo2C toward the reverse water–gas shift reaction
Source: Front Chem. 2023 Mar 20;11:1144189. doi: 10.3389/fchem.2023.1144189 (PMC10067905; doi:10.3389/fchem.2023.1144189)
Supplement: Supplementary file 1 [file DataSheet1.docx]

**Supporting Information**

**Theoretical Study of the Catalytic Performance of Fe and Cu Single-Atom Catalysts Supported on Mo_2_C towards the Reverse Water-Gas Shift Reaction (RWGS)**

Wenjuan Zhang^1,†^, Anna Vidal-López^1,†^, and Aleix Comas-Vives^1,2,*^

*^1^Department of Chemistry, Universitat Autònoma de Barcelona, 08193 Cerdanyola del Vallès, Catalonia, Spain*

*^2^Institute of Materials Chemistry, Technische Universität Wien, 1060 Vienna, Austria*

^†^These authors contributed equally to this work and share first authorship

Corresponding author: Prof. Dr. Aleix Comas Vives^*^ ([Aleix.Comas@uab.cat](mailto:Aleix.Comas@uab.cat), aleix.comas@tuwien.ac.at)

- *Tables of computed adsorption and desorption energies*

**Table S1.** Adsorption energies of O, H, CO_2,_ and CO for M(Cu/Fe)@Mo_2_C with 0 O* ML defined as E[ads]= E[X*]–E[X]–E[*], where (X) is the atom/molecule, (X*) is the atom/molecule adsorbed and (*) is the substrate. Energies are in eV.

|  | **Fe/Mo_2_C** | | | | **Cu/Mo_2_C** | | | |
| --- | --- | --- | --- | --- | --- | --- | --- | --- |
| **Adsorbent** | **Top** | **Bridge** | **H_x_** | **H_m_** | **Top** | **Bridge** | **H_x_** | **H_m_** |
| **O** | -1.93 | -1.54 | -2.41 | -1.99 | -1.41 | -1.66 | -2.46 | -2.04 |
| **H** | -0.73 | -0.63 | -0.68 | -0.7 | -0.19 | -0.62 | -0.69 | -0.68 |
| **CO_2_** | -0.84 | -1.97 | -1.78 | -1.73 | -0.49 | -1.15 | -1.43 | -1.39 |
| **CO** | -2.55 | -1.99 | -1.97 | -1.92 | -2.07 | -2.05 | -2.04 | -1.97 |

**Table S2.** CO* desorption energies on Fe/Mo_2_C system for each coverage in kJ mol^-1^ without the Fe motion energy. This energy has been only calculated for the 0 and 0.33 O* ML and its show in parentheses.

| **kJ mol^-1^** | **0 O*** | **0.33 O*** | **0.67 O*** | **0.78 O*** |
| --- | --- | --- | --- | --- |
| **CO* desorption** | 125.1 (23.6) | 135.6 (31.4) | 93.9 | 120.7 |

- *Geometries of Optimized Structures (Intermediates and TS) of the preferred reaction path*

*
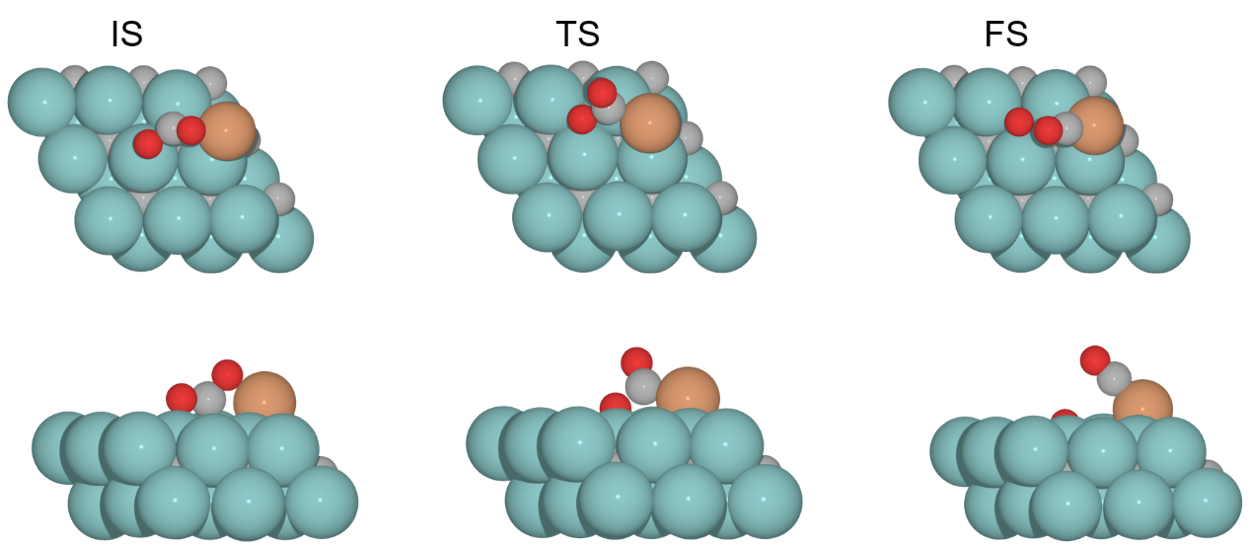
*

**Figure S1**. Top and side views of the initial state (**IS**), transition state (**TS**), and final state (**FS**) for the CO_2_ cleavage catalyzed by the Fe/Mo_2_C of 0 ML system.


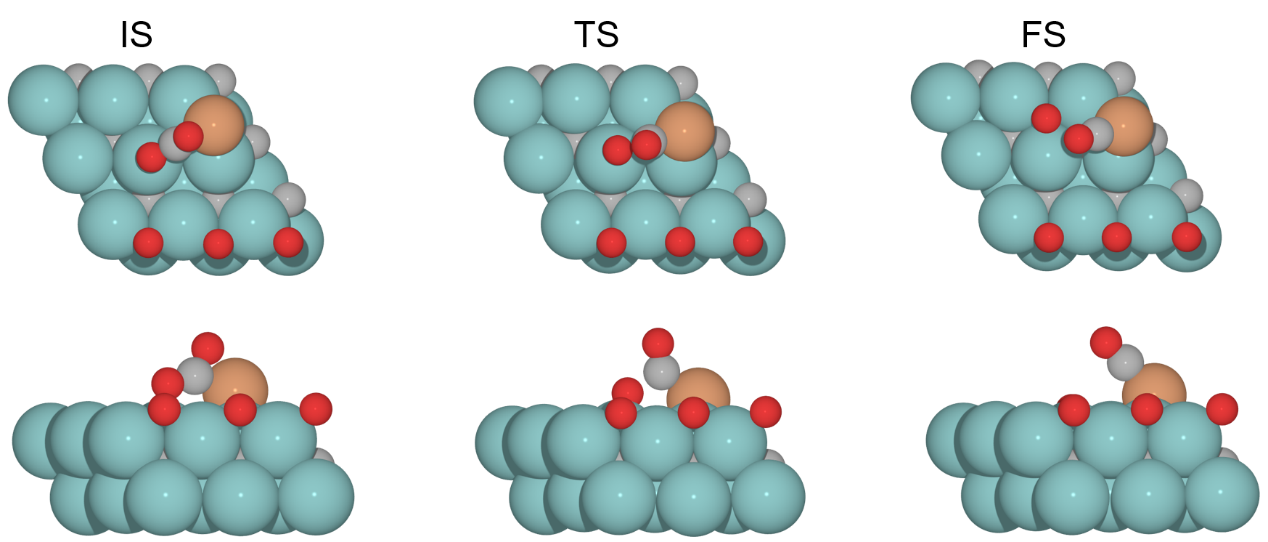


**Figure S2**. Top and side views of the initial state (**IS**), transition state (**TS**), and final state (**FS**) for the CO_2_ cleavage catalyzed by the Fe/Mo_2_C of 0.33 ML system.


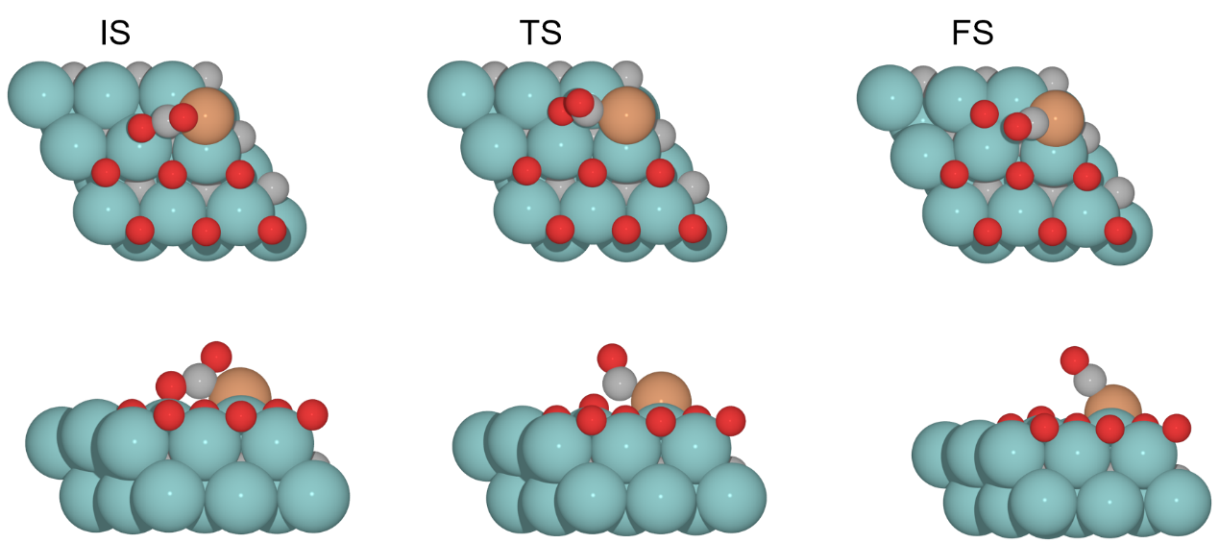


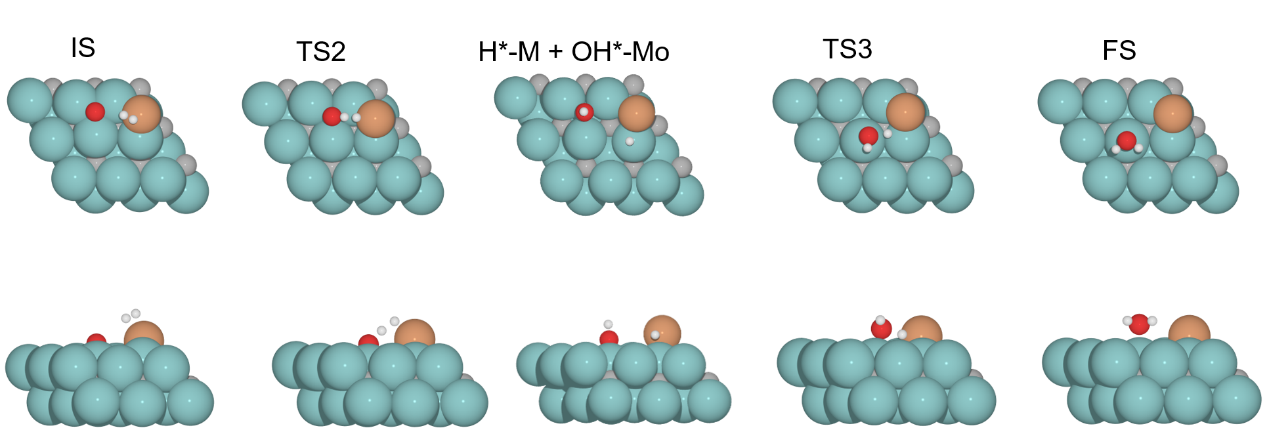
**Figure S3**. Top and side views of the initial state (**IS**), transition state (**TS**), and final state (**FS**) for the CO_2_ cleavage catalyzed by the Fe/Mo_2_C of 0.67 ML system.

**Figure S4**. Top and side views of the initial state (**IS**), transition state (**TS**), and final state (**FS**) for H_2_ splitting, and formation of H_2_O catalyzed by the Fe/Mo_2_C of 0 ML.


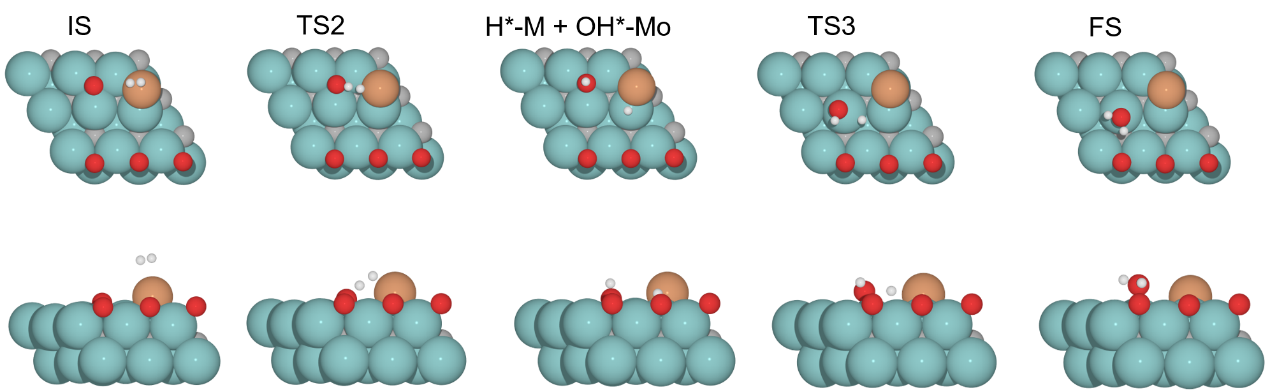


**Figure S5**. Top and side views of the initial state (**IS**), transition state (**TS**), and final state (**FS**) for H_2_ splitting and formation of H_2_O catalyzed by the Fe/Mo_2_C of 0.33 ML.

**
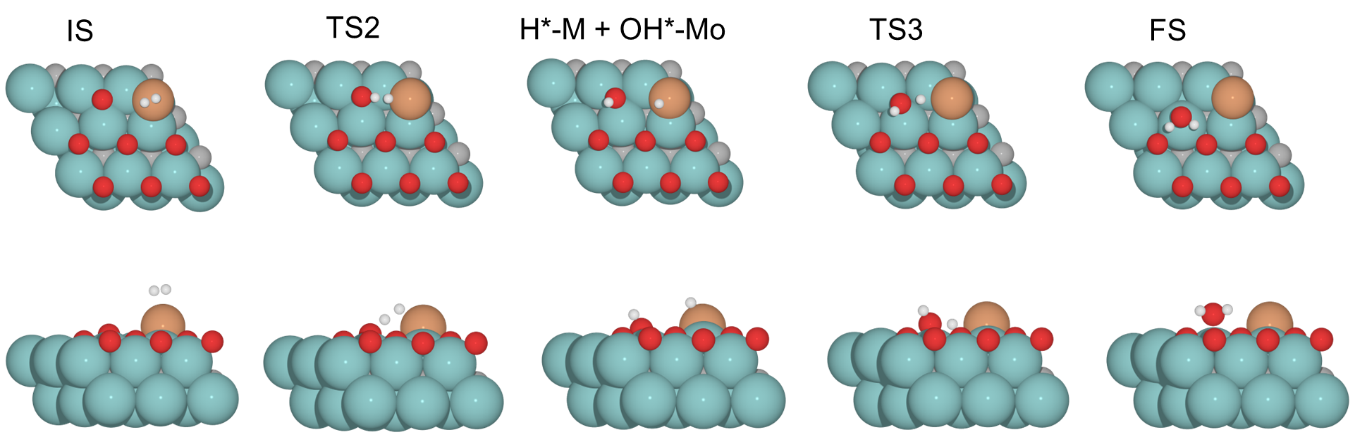
**

**Figure S6**. Top and side views of the initial state (**IS**), transition state (**TS**), and final state (**FS**) for H_2_ splitting and formation of H_2_O catalyzed by the Fe/Mo_2_C of 0.67 ML.


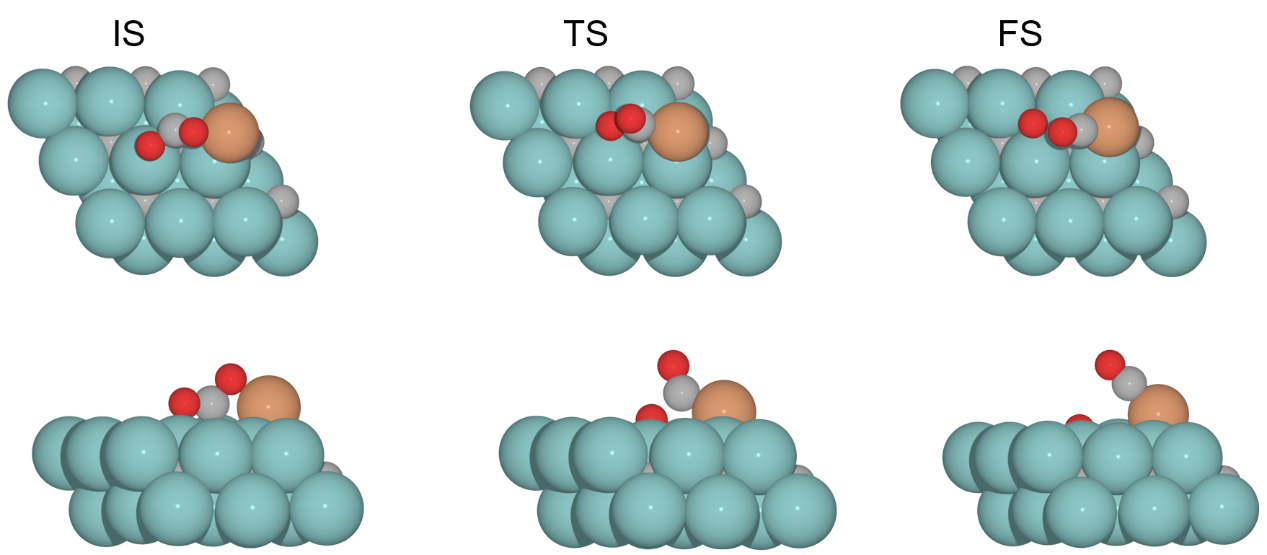


**Figure S7**. Top and side views of the initial state (**IS**), transition state (**TS**), and final state (**FS**) for the CO_2_ cleavage catalyzed by the Cu/Mo_2_C of 0 ML system.


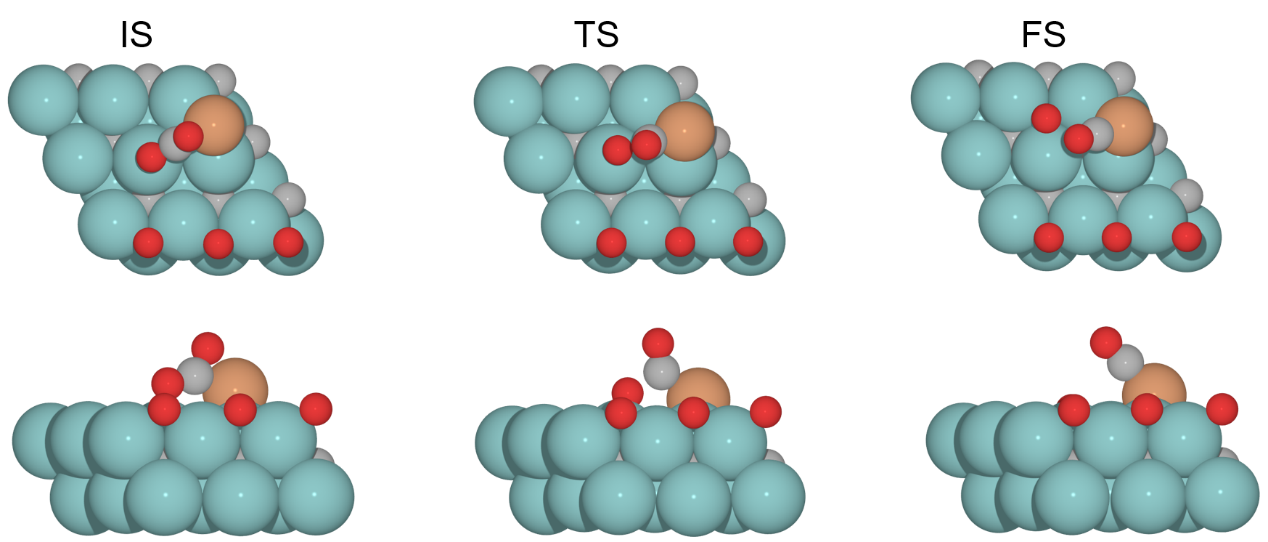


**Figure S8**. Top and side views of the initial state (**IS**), transition state (**TS**), and final state (**FS**) for the CO_2_ cleavage catalyzed by the Cu/Mo_2_C of 0.33 ML system.


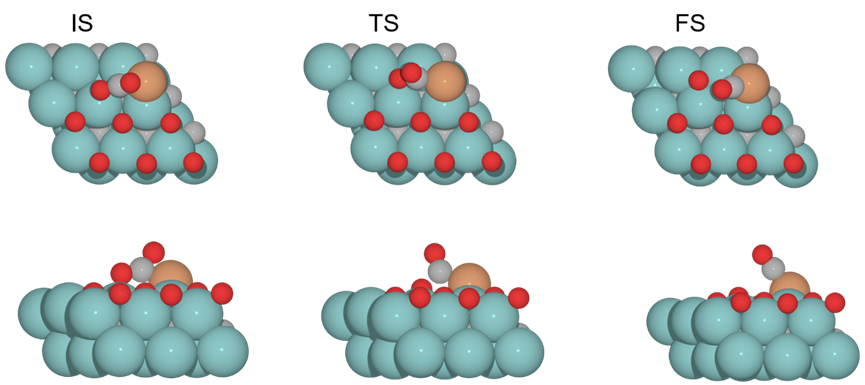


**Figure S9**. Top and side views of the initial state (**IS**), transition state (**TS**), and final state (**FS**) for the CO_2_ cleavage catalyzed by the Cu/Mo_2_C of 0.67 ML system.


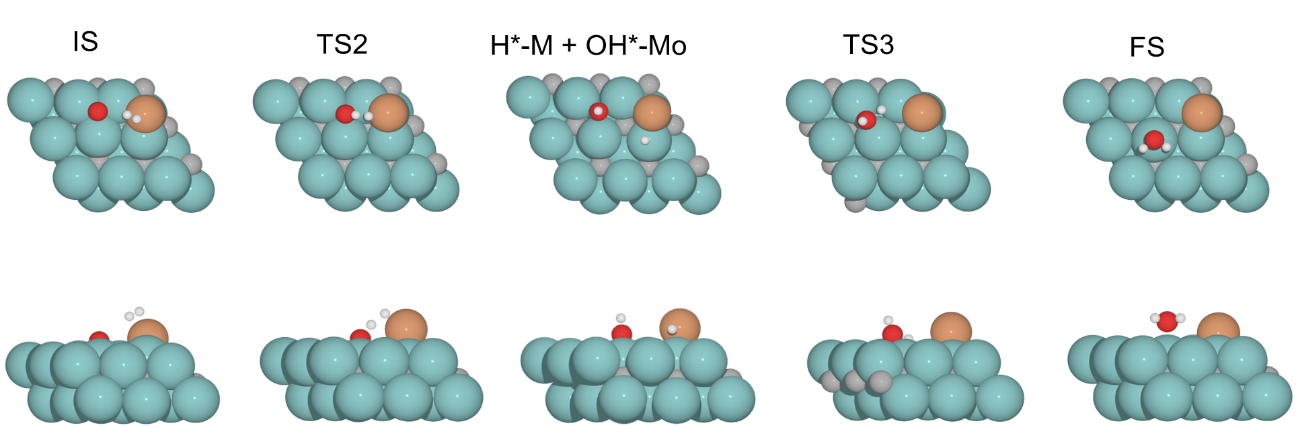


**Figure S10**. Top and side views of the initial state (**IS**), transition state (**TS**), and final state (**FS**) for

H_2_ splitting and formation of H_2_O catalyzed by the Cu/Mo_2_C of 0 ML.


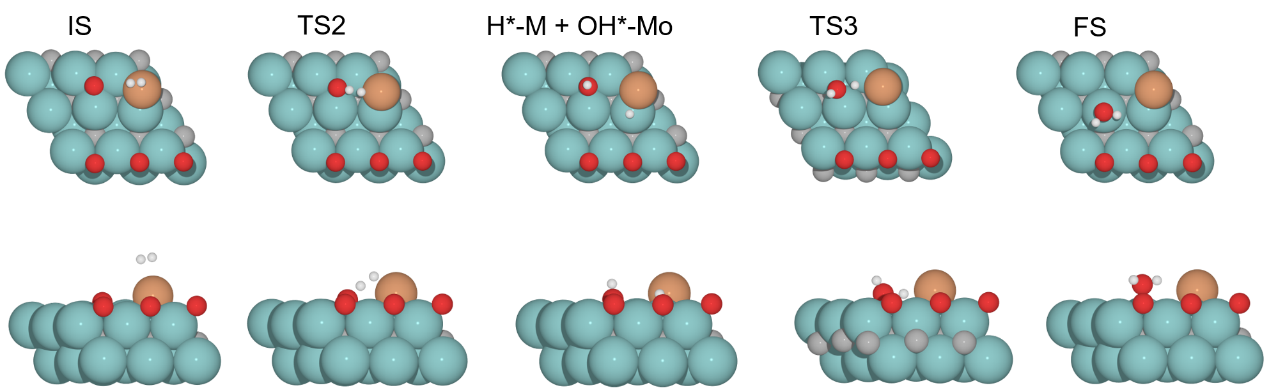


**Figure S11**. Top and side views of the initial state (**IS**), transition state (**TS**), and final state (**FS**) for H_2_ splitting and formation of H_2_O catalyzed by the Cu/Mo_2_C of 0.33 ML.


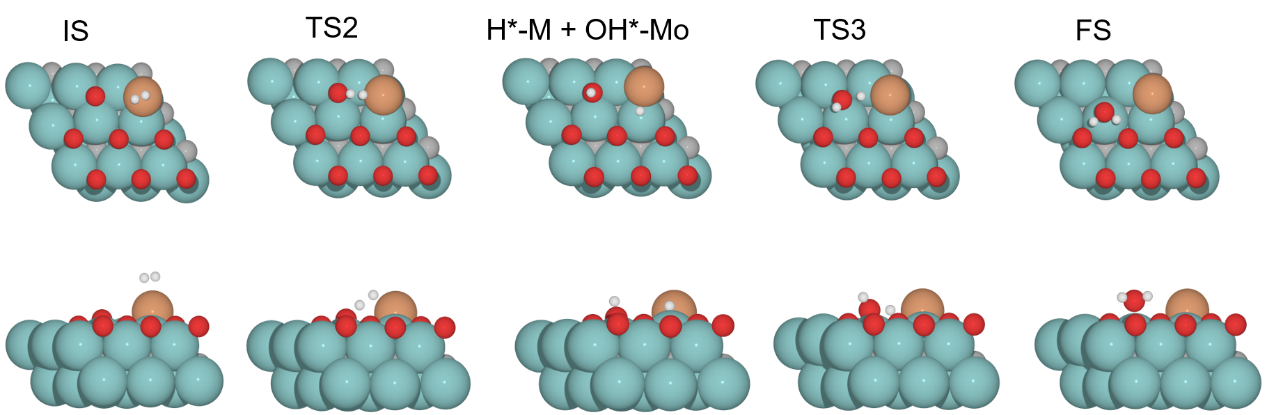


**Figure S12**. Top and side views of the initial state (**IS**), transition state (**TS**), and final state (**FS**) for H_2_ splitting and formation of H_2_O catalyzed by the Cu/Mo_2_C of 0.67 ML.
